# Enhanced Recovery After Surgery (ERAS) Reduces Hospital Costs and Length of Hospital Stay in Radical Cystectomy: A Prospective Randomized Controlled Study

**DOI:** 10.7759/cureus.55460

**Published:** 2024-03-03

**Authors:** Buğra Çetin, Nusret Can Çilesiz, Arif Ozkan, Özkan Onuk, Gülay Kır, M B Can Balci, Enver Özdemir

**Affiliations:** 1 Urology, Altınbaş University Medicalpark Bahçelievler Hospital, Istanbul, TUR; 2 Urology, Biruni University Hospital, Istanbul, TUR; 3 Urology, Koç University, Istanbul, TUR; 4 Anesthesiology and Reanimation, Koç University, Istanbul, TUR; 5 Urology, Taksim Training and Research Hospital, Istanbul, TUR; 6 Urology, Gaziosmanpaşa Training and Research Hospital, Istanbul, TUR

**Keywords:** length of hospital stay (los), cost of hospitalization, radical cystectomy, bladder cancer, eras

## Abstract

Enhanced recovery after surgery (ERAS) protocols challenge the conventional and rigid methods of surgery and anesthesia and bring about novel changes that are quite drastic. The core principle of the protocol is to minimize the metabolic disturbance caused by surgical injury, facilitate the restoration of functions in a brief period, and promote the resumption of normal activity at the earliest. To compare the outcomes of ERAS and standard protocol for patients who have undergone radical cystectomy (RC) with ileal conduit urinary diversion. This prospective randomized controlled study was performed between 2015 and 2023. The 77 patients were divided into two groups ERAS (n=39) and Standard (n=38) by sequential randomization. These two groups are divided according to protocols of bowel preparation, anesthesia, and postoperative nutrition. The clinical and demographic characteristics of the patients, and the American Society of Anesthesiologists (ASA) and Eastern Cooperative Oncology Group (ECOG) scores were recorded. Perioperative findings, the degree of complications according to the Clavien-Dindo classification, and the total cost of treatment were recorded and analyzed. Length of hospital stay (18.82±9.25 day vs 27.34±15.05 day), and cost of treatment (2168,2±933$ 2879±1806$) were higher in the standard group. The rate of nausea and vomiting and the use of antiemetics were higher in the ERAS group compared to the standard group. In patients undergoing RC, the ERAS protocol was found to shorten the duration of hospitalization and reduce the total cost of hospital stay.

## Introduction

The standard treatment for muscle-invasive bladder cancer (MIBC) is radical cystectomy (RC). Cystectomy and pelvic lymph node dissection provide the best cancer-specific survival in MIBC [1]. RC provides excellent local control in lymph node-negative patients with a local recurrence rate of 4% [2].

RC is a major surgical procedure with high complication and mortality rates. Urinary diversion is one of the most important parts of this surgery and bowel segments are generally used for this. Serious postoperative complications can be seen due to bowel resection and anastomosis. The rate of complications has been found to be 13%, and the mortality rate within 30 days 1.5% [3]. Although the mortality rate of RC has decreased in the past decade, early morbidity rates have remained at 11-68% [4,5].

Enhanced recovery after surgery (ERAS) is a term used to describe multimodal and begins in the outpatient stage and then includes preoperative and perioperative procedures to improve perioperative outcomes. ERAS allows the acceleration of postoperative recovery, decreases hospital stay (two to three days), and reduces morbidity (30%-50%) [6].

The main purpose of the ERAS protocol is to reduce the metabolic stress caused by radical surgery. Despite many studies in the literature, the ERAS protocol is a new application in the field of urology. Approximately 20 studies have been conducted with the ERAS protocol in RC, but there are only a few prospective studies that compare the standard protocol and ERAS in terms of postoperative complications, length of hospital stay, and cost. We aimed to compare the length of hospital stay, the postoperative early complications and the total costs of the patients who underwent RC and ileal loop urinary diversion, between the ERAS and standard protocol.

## Materials and methods

Participants

The prospective randomized controlled study was conducted after approval from the Taksim Training and Research Hospital Ethics Committee at the Gaziosmanpaşa Taksim Training and Research Hospital Urology Clinic, between January 2015 and November 2023. The patients who had undergone open RC with ileal loop urinary diversion for MIBC were included in the study. Patients who had received neo-adjuvant chemotherapy for bladder cancer, insulin-dependent diabetic patients, patients with neurological comorbidities, patients with other malignancies, patients who had undergone another urinary diversion technique other than the ileal loop, and patients with gastrointestinal system disease or previous gastrointestinal surgery were excluded from the study.

Cost calculations were specifically conducted based on care fees and medication costs. Operating room expenses and surgical fees were not included in these calculations, as they were not within the scope of the study's cost analysis.

The patients were divided into two groups: the ERAS group (n=39) and the standard protocol group (n=38) by sequential randomization. The clinical and demographic characteristics of the patients (age, gender, medical and family history, body mass index, and the American Society of Anesthesiologists (ASA) and Eastern Cooperative Oncology Group (ECOG) scores were recorded.

**Figure 1:**
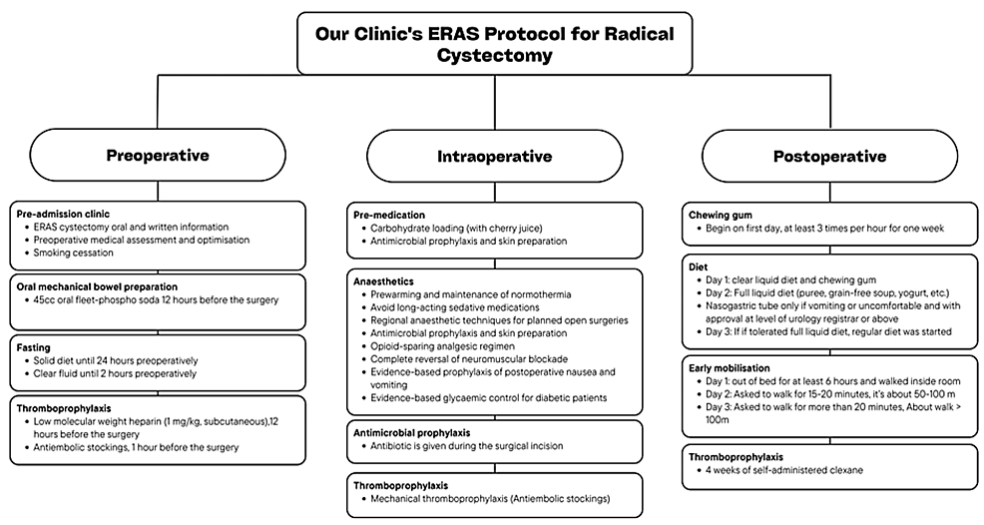
Our clinic’s eras protocol for radical cystectomy Image credits: Buğra Çetin

Standard anesthesia protocol was applied to the patients by an anesthesiologist during the pre-operative period. In all patients, unnecessary opioid use was avoided in the peri-operative and postoperative period, thanks to the analgesia provided by the epidural catheter applied through the appropriate vertebral space (lumbar/thoracic). In the pre-operative period, short-acting opioids, such as remifentanil, were used instead of long-acting opioids. Controlled hypotension was administered to reduce peri-operative bleeding. Fluid balance was closely monitored, and hypovolemia and fluid overload were avoided to prevent splanchnic hypoperfusion (Figure 1).

ERAS Protocol

The patients were given breakfast at 06:00 the morning the day before the surgery and a clear liquid diet (water, clear apple juice, plain tea without milk or milk substitute) was applied until two hours prior to our scheduled arrival time of the surgery. The patients weren’t given any specific carbohydrate-loading drink pre-operatively. The patients were not given any intravenous fluid support until the morning of the surgery. The patients were given 45cc oral fleet-phospho soda 12 hours before the surgery. Low molecular weight heparin (1 mg/kg, subcutaneous) was started 12 hours before the surgery and continued for four weeks. Antiembolic stockings, which were put on the patients by health personnel 1 hour before the operation, were used during hospitalization. Epidural analgesia was administered to the patients where there were no contraindications (Figure 1).

Patients who underwent the ERAS protocol were mobilized on the first day after surgery. The patients were switched to a clear liquid diet of 30-60 mL per hour without waiting for flatus or defecation and additional intravenous fluid supplementation (100cc/hr Dextrose Monohydrate 5g) was given. To increase the bowel movements of the patients, sugarless chewing gum was chewed at least three times per hour for one week, and chewing gum was chewed daily. A full liquid diet ((puree, grain-free soup, yogurt, etc.)) was started on the second postoperative day, if tolerated, a regular diet was started one day later. Maintenance of intravenous fluid was continued at 60cc per hour. Intravenous fluid was stopped when a regular diet was tolerated (Figure 1).

Standard Protocol

Regular diet restriction was applied for three days pre-operatively in patients following the standard protocol and only a clear liquid diet was given. Oral intake was stopped the night before surgery until post-operative flatulence. A nasogastric tube was inserted routinely before surgery and kept in place until the 2nd postoperative day (Figure 1). No recommendation was made to the patients about chewing gum. A clear liquid diet was started when patients passed flatus, and the normal diet was changed in turn if the regimen was fully tolerated. If there were no contraindications to provide analgesia, epidural analgesia was performed, and opioid derivatives were used if necessary. 

Measures

For the patients in both groups, pre-operative and post-operative 1st and 7th-day creatinine, albumin, and hemoglobin values ​​, operation time, peri-operative bleeding amount, whether blood transfusion was performed, time of flatus or defecation, the time needed for oral food toleration, the need for an antiemetic and/or nasogastric tube, the complication degrees according to the Clavien-Dindo classification, the length of hospital stay, and the total cost of treatment were recorded.

Procedures

Following the RC perioperative guidelines of the ERAS Society (2013), Western Health developed and implemented an ERAS protocol in June 2019. This protocol was the result of extensive consultation with key stakeholders who are involved in the care of cystectomy patients at Western Health, such as Stomal Therapy, Urology Nursing, Pre-Admission Clinic, Allied Health, Anaesthetics, Pharmacy, Intensive Care, and Pain Services. The protocol consists of three phases: preoperative, intraoperative, and postoperative (Figure 1) [6].

Statistical method

As a result of the power analysis, it was determined that the sample size should be at least 77 patients at an acceptable 5% error and 95% confidence level. The data of the 77 patients were analyzed using the IBM SPSS Statistics 23 program (IBM Corp., Armonk, NY). While evaluating the study data, parametric tests were used by applying the proposition that the distribution of the sample mean approaches the normal distribution for numerical variables, n→∞ according to the Law of Large Numbers (İNAL and GÜNAY, 2002, p. 264). Frequency distribution for categorical variables and descriptive statistics (mean, standard deviation, minimum, maximum) for numerical variables are given below. The “significance test of the difference between two means” (independent t-test) was used to examine the differences between categorical variables of the two groups, and the Chi-square test was used to examine the relationship between two categorical variables. The results are given in the tables in the following section.

## Results

Seventy-seven patients were included in this study. The demographic properties of the patients have been shown in Table 1. An open RC operation was performed on 39 of these patients following the ERAS protocol and 38 with the standard protocol. There were no significant differences between the two groups in terms of, BMI, operation time, time of defecation, creatinine, preop albumin, post-op early albumin, and pre-discharge albumin values. ​​ 

**Table 1:** Measured parameters in patients undergoing radical cystectomy in ERAS vs standard treatment groups

		ERAS (n=39)	Standard (n=38)	P-value
BMI, kg/m2 (Mean ± std)		23.58 ± 3.511	24.00 ± 2.672	0.562
Operation duration, min (Mean ± std)		352.82 ± 56.566	363.68 ± 67.320	0.445
					Blood loss, mL (Mean ± std)		377.18 ± 276.195	575.79 ± 324.834	0.005
Preoperative minus postoperative Hb value, g/dL (Mean ± std)		2.58 ± 0.963	2.7 ± 0.960	0.028
					Time of first flatulence, hr (Mean ± std)		37.92 ±15.426	56.58 ± 19.260	<0.001
Time of first defecation , day (Mean ± std)		5.97 ± 14.833	5.45 ± 1.537	0.828
Full tolerance to oral intake, day (Mean ± std)		4.18 ± 1.520	5.68 ± 1.890	<0.001
					Hospitalisation duration, day (Mean ± std)		18.82 ± 9.250	27.24 ± 15.057	0.004
Cost of treatment without surgery (US $)		2168.2 ± 933	2879 ± 1806	0.032					
Creatinine, mg/dL (Mean ± std)	preop	1.6 ± 2.902	1.54 ± 2.392	0.913
					postop	1.22 ± 0.512	1.30 ± 0.762	0.576
	Albumin, g/dL (Mean ± std)	preop	3.20 ± 0.667	3.22 ± 0.611	0.880
postop						2.68 ± 0.336	2.53 ± 0.421	0.082

The mean Hb difference (1.58±0.96 vs 2.07±0.96; p=0.028), length of hospital stay (18.82±9.25 vs 27.34±15.05; p=0.004), and total cost of treatment (2,168, 2±933US$; 2879±1806US$; p=0.032) were higher in the standard group compared to the ERAS group. 

Time of the flatulence (h) (37.92±15.42 vs 56,58±19.26 hours; p<0.001) and time to achieve tolerance of oral nutrition (4.18±1.5 vs 5.68±1.8 day; p<0.001) after RC and urinary diversion with ileal loop were found lower significantly in the ERAS group (Table 1).

The rate of nausea and vomiting (59% vs 23.6% p=0.002) and requirement of antiemetics (59% vs 23.7% p=0.002) were higher in the ERAS group but ileus (% 20.5 vs %42.1; p=0.041), and NG catheterization (20.5% vs 42.1%; p=0.041) were lower in the ERAS group (Table 2).

**Table 2:** Clavien complication rates in patients undergoing radical cystectomy in ERAS vs standard treatment groups

	ERAS Group (N=39)	Standard Group (N=38)	P-value
Clavien I	37 (94.8%)	36 (94.7%)	0.978
Nausea-Vomiting	23 (59.0%)	9 (23.6%)	0.005
Ileus	8 (20.5%)	16 (42.1%)	0.035
Urinary Infection	N/A	N/A	
Wound Infection	3 (7.7%)	4 (10.5%)	0.663
Ureteral Catheter Obstruction	N/A	N/A	
Intraabdominal Urine Leakage	3 (7.7%)	7 (18.4%)	0.285
Tolerated anemia	N/A	N/A	
Clavien II	12 (%30.1)	16 (%42.1)	0.301
Anemia that requires transfusion	9 (23.1%)	14 (36.8%)	0.180
Pulmonary Embolism	N/A	2 (5.1%)	0.239
Pyelonephritis	2 (5.1%)	2 (5.1%)	0.978
Confusion or Neurological Disease	N/A	1 (2.6%)	0.494
Pneumonia	1 (2.6%)	3 (7.7%)	0.292
Clavien III	2 (5.1%)	4 (10.5%)	0.377
Ureteral Reflux	N/A	N/A	
Compressive Lenfocele	N/A	N/A	
Ileal anastomosis leakage	1 (2.6%)	2 (5.1%)	0.541
Evisceration	1 (2.6%)	2 (5.1%)	0.541
Clavien IV	0	3 (7.9%)	0.115
Rectal Necrosis	N/A	N/A	
Loop-Neobladder rupture	N/A	N/A	
Severe sepsis	N/A	1 (2.6%)	0.494
Nonobstructive renal failure	N/A	2 (5.1%)	0.240
Single Organ Failure	N/A	N/A	
Multi organ failure	N/A	N/A	
Clavien V	1	5 (13.2%)	0.829
Death	1 (2.6%)	5 (13.2%)	0.829

According to the degree of Clavien-Dindo classification, there was no difference in the rate of complications (I-V) between the two groups (Table 3).

**Table 3:** Examination of the relationship between demographic and clinical information and protocol types

		ERAS (n(%))	Standard (n(%))	P-value
Gender	Male	36(92.3)	33 (86.8)	0.481
	Female	3 (7.7)	5 (13.2)	-
ECOG PS	0	14 (35.9)	17 (44.7)	-
	1	23 (59.0)	19 (50.0)	-
	2	2 (5.1)	2 (5.3)	-
Smoking	Smoker	13 (33.3)	9 (23.7)	0.349
	Non-smoker	26 (66.7)	29 (76.3)	
ASA	1	12 (30.8)	9 (23.7)	0.398
	2	23 (59.0)	21 (55.3)	
	3	4 (10.3)	8 (21.1)	
Blood Transfusion		17 (43.6)	19 (50.0)	0.573
Antiemetic Need		23(59.0)	9 (23.7)	0.002
NG catheterisation		8 (20.5)	16 (42.1)	0.041
Ileus		8 (20.5)	16 (42.1)	0.041
Nausea- Vomiting		23 (59.0)	9 (23.6)	0.002
Clavien Complication	1	37(94.8)	36 (94.7)	0.978
	2	12(30.8)	16 (42.1)	0.301
	3	2 (5.1)	4 (10.3)	0.377
	4	0	3(7.9)	0.115
	5	1(2.6)	5 (13.2)	0.108

## Discussion

Difference between length of hospital stay

The ERAS protocol has been used for two decades in colorectal surgeries, and a decrease is observed in parameters such as postoperative complications and hospital stay compared to the standard protocol [7-11]. Several prospective randomized studies regarding postoperative complications and hospital stay for ERAS and standard nutrition protocols comparing an RC with ileal loop urinary diversion have been published [12-17]. The main differences that separate the ERAS protocol from the standard are preoperative counseling of the patient, a carbohydrate-rich diet until the early preoperative period, controlled perioperative fluid management, ileus management differences (gum, metoclopramide) [12], early mobilization, and early oral intake [12].

Many studies have shown that the length of hospital stay is lower with the ERAS protocol [18-20]. Mukhtar et al. [21], reported that although the mean length of hospital stay was one day less in the ERAS group, it was not statistically significant. In our study, it was shown that the duration of hospital stay was reduced for patients who followed the ERAS protocol (18.82±9.25) compared to the patients who followed the standard protocol (27.34±15.05), and it was statistically significant (p=0.004).

Our average hospital stay was longer compared to the literature [19,20]. In the Turkish population, we suggested some subjective criteria causing a long duration of hospital stay such as crowded house population, having anxiety about what they cannot do about urostomy care, and feeling that it will be difficult for them to reach the hospital if they have a problem. These issues can lead to the patient not wanting to be discharged without feeling completely well and so affect the duration of the stay in the hospital.

According to the guidelines for RC and ERAS recommendation, the first item is pre-operative counseling and education. Fully informing the patients who are ready for discharge about the next steps can reduce their anxiety and thus decrease the length of stay in the hospital. This is one of the reasons why the ERAS protocol has a shorter hospital stay than the standard protocol.

Difference between minor complications

In some studies [21,22], the duration of bloating observed in patients was noted. In contrast, in our study, the time to first flatulence, the day of defecation, and the day when regular diet could be tolerated were recorded. Recent meta-analyses have shown that the time to first flatulence is earlier in patients who have undergone ERAS after RC in patients with MIBC [17]. Similar to the literature, time to first flatulence and regular diet tolerability were earlier in patients treated with the ERAS protocol compared to patients on the standard protocol in this study. When the first day of defecation was compared, it was shown that there was no significant difference between the two groups.

Studies have shown that nausea and vomiting in the initial period are more common in patients who underwent the ERAS protocol [21]. After the postoperative day 3, it has been shown that nausea is more common in patients with the standard protocol [12]. In our study, the need for post-operative antiemetics was higher in the ERAS protocol group than in the standard protocol group. This may be due to the early transition to a regular diet after the surgery. It was observed that the need for a nasogastric tube was higher in patients with the standard protocol. The reason for this is that nothing by mouth (NPO), for a long time. It may delay the onset of normal bowel functions in patients which in turn leads to the development of paralytic ileus.

In the meta-analysis published by Tyson et al. [17], it was shown that the ERAS protocol reduced the length of hospital stay, overall complication rate, and the time to reach normal bowel function compared to the standard protocol. Frees et al. [12] demonstrated that there was no significant difference in the overall complication rates. Guan et al. [18], reported a decrease in overall complication rates with the ERAS protocol. In the remaining 10 studies in the literature, no significant difference was found between the rates of complications encountered in the early postoperative period between ERAS and standard protocol applications. In our study, it was observed that the complication rates were similar in both protocols and no significant difference was observed.

Difference between the cost of treatment

In the study conducted by Nabhadi. et al. [23], the costs of patients who followed the ERAS protocol and standard protocol were compared, and it was shown that the average cost of the patients following ERAS was US$ 4,495 less. Similarly, in our study, it was shown that the average cost of the patients who underwent the standard protocol was significantly higher than the patients who underwent the ERAS protocol. The cost reduction is due to the shorter average hospital stay and fewer intestinal complications, such as ileus.

Difference between complications during the surgery

Frees et al. [12] found that there was no significant difference between ERAS and the standard protocol in terms of the amount of bleeding during the surgery. In our study, the amount of peri-operative bleeding was higher in the patients who underwent the standard protocol than in the patients on the ERAS protocol. The reason for this is related to the hypotensive anesthesia protocol applied to the ERAS group.

Limitations

The limitations of our study were that different surgical teams carried out the surgeries and that it covered a relatively small number of patients. The other limitation is the lack of pre-operative specific carbohydrate-loading drinks and immunonutrition. Another limitation is; that we did not have an objective criterion for measuring the malnutrition levels of the patients before the procedure. The only value we investigated for this was albumin, not pre-albumin.

## Conclusions

In summary, our study comparing ERAS protocol with the standard approach for RC in MIBC patients revealed significant advantages with ERAS. Despite higher rates of nausea and vomiting, the ERAS group exhibited a notable reduction in the length of hospital stay, early complications, and overall treatment costs. Patients following ERAS demonstrated faster recovery milestones, including a quicker return of bowel function and oral nutrition tolerance.

Financially, ERAS proved to be more cost-effective, largely attributed to a shorter hospital stay and decreased postoperative complications. While acknowledging study limitations, such as variations in surgical teams and a modest sample size, our findings underscore the potential benefits of implementing ERAS in urological practices, emphasizing the importance of patient education and personalized care pathways.
